# The 11q Terminal Deletion Disorder Jacobsen Syndrome is a Syndromic Primary Immunodeficiency

**DOI:** 10.1007/s10875-015-0211-z

**Published:** 2015-11-14

**Authors:** Virgil A. S. H. Dalm, Gertjan J. A. Driessen, Barbara H. Barendregt, Petrus M. van Hagen, Mirjam van der Burg

**Affiliations:** Department of Internal Medicine, Erasmus MC, ‘s-Gravendijkwal 230, 3015 CE Rotterdam, The Netherlands; Department of Immunology, Erasmus MC, ‘s-Gravendijkwal 230, 3015 CE Rotterdam, The Netherlands; Department of Pediatric Infectious disease and Immunology, Erasmus MC, ‘s-Gravendijkwal 230, 3015 CE Rotterdam, The Netherlands

**Keywords:** Immunodeficiency, Jacobsen syndrome, 11q terminal deletion disorder, humoral, infections

## Abstract

**Background:**

Jacobsen syndrome (JS) is a rare contiguous gene syndrome caused by partial deletion of the long arm of chromosome 11. Clinical features include physical and mental growth retardation, facial dysmorphism, thrombocytopenia, impaired platelet function and pancytopenia. In case reports, recurrent infections and impaired immune cell function compatible with immunodeficiency were described. However, Jacobsen syndrome has not been recognized as an established syndromic primary immunodeficiency.

**Goal:**

To evaluate the presence of immunodeficiency in a series of 6 patients with JS.

**Methods:**

Medical history of 6 patients with JS was evaluated for recurrent infections. IgG, IgA, IgM and specific antibodies against *S. pneumoniae* were measured. Response to immunization with a polysaccharide vaccine (Pneumovax) was measured and B and T lymphocyte subset analyses were performed using flowcytometry.

**Results:**

Five out of 6 patients suffered from recurrent infections. These patients had low IgG levels and impaired response to *S. pneumoniae* polysaccharide vaccination. Moreover, we also found a significant decrease in the absolute number of memory B cells, suggesting a defective germinal center function. In a number of patients, low numbers of T lymphocytes and NK cells were found.

**Conclusions:**

Most patients with JS suffer from combined immunodeficiency in the presence of recurrent infections. Therefore, we consider JS a syndromic primary immunodeficiency. Early detection of immunodeficiency may reduce the frequency and severity of infections. All JS patients should therefore undergo immunological evaluation. Future studies in a larger cohort of patients will more precisely define the pathophysiology of the immunodeficiency in JS.

**Electronic supplementary material:**

The online version of this article (doi:10.1007/s10875-015-0211-z) contains supplementary material, which is available to authorized users.

## Introduction

Jacobsen syndrome (JS) is a contiguous gene syndrome caused by partial deletion of the long arm of chromosome 11, and was initially described by the Danish physician dr. Petra Jacobsen in 1973 [1]. It is a rare disorder with estimated occurrence of about 1/100,000 births and a female to male ratio of 2:1 [2–4]. JS is caused by partial deletions of the long arm of chromosome 11, del(11)(q23) [1]. The deletion size ranges from 5 to 20 Mb. Breakpoints typically arise within or distal to subband 11q23.3 and the deletion usually extends to the telomere. Partial JS with a 5 Mb deletion has been described as well [2, 5].

The most common clinical features include pre- and postnatal physical growth retardation, psychomotor retardation, behavioural changes, characteristic facial dysmorphism and thrombocytopenia/thrombocyte dysfunction (Paris-Trousseau thrombocytopenia with dysmegakaryopoiesis) or pancytopenia. A subset of patients has malformations of the heart, kidney, gastrointestinal tract, genitalia, central nervous system and/or skeleton. Ocular, hearing and endocrine problems may be present as well [2, 3, 6, 7].

Although JS has not been recognized as a syndromic primary immune deficiency (SPID) so far, recurrent episodes of otitis media and/or sinusitis were described in 42 of 78 subjects (54 %) in a prospective study in patients with JS. At that time, serum IgA levels were evaluated in 13 subjects and were found to be normal or low normal for age. No other immunodeficiencies were evaluated in this study [2]. In previous years, several case reports have described the presence of an immunodeficiency state in patients with JS. The most important findings in these cases are summarized in Table 1 [8–12]. Clinical and laboratory features of patient 2,3 and 4 in Table 1 consist of a reduction of all immunoglobulin subtypes (IgG, IgA and IgM) and impaired response to pneumococcal polysaccharide vaccination. These features are compatible with the primary antibody deficiency common variable immunodeficiency (CVID). Current diagnostic features for CVID, according to the European Society for Immunodeficiencies (ESID)/Pan American Group for Immune Deficiency (PAGID) (1999), ESID (2014) and new diagnostic criteria proposed by Ameratunga et al. [13] for CVID, include reduction in levels of serum IgG in combination with low levels of IgA with or without low levels of IgM, poor or absent response to immunizations and/or low number of switched memory B cells and an absence of any other defined immunodeficiency states [14]. Apart from recurrent sinopulmonary tract and gastro-intestinal tract infections, CVID may also be associated with the development of auto-immune phenomena and (haematological) malignancies [15]. Clinical features and additional findings of patients 1, 5 and 6 in Table 1 point towards a combined immunodeficiency, in which B and T lymphocyte and NK cell functions seem to be impaired.

**Table 1:** Described immune deficiencies in patients with Jacobsen syndrome

	Patient 1 ♀ 12 years [8]	Patient 2 ♂ 12 years [9]	Patient 3 ♂ 4 years [10]	Patient 4 ♂ 5 years [10]	Patient 5 ♂ 34 years [11]	Patient 6 ♀ 45 years [12]
Clinical features	Recurrent respiratory tract infections	Chronic diarrhea, recurrent respiratory tract infections	Recurrent respiratory tract infections, otitis media, sinusitis	Enterobacter Cloacae mediastinitis, Klebsiella tracheitis, CNS bacteremia	Increased incidence of viral and bacterial infections	Recurrent pneumococcal pneumoniae, genital and cutaneous condylomata
IgG	5,4 g/L (normal)	**2,26 g/l** (low)	**2,81 g/l** (low)	**1,61 g/l** (low)	Not performed	**3,4 g/l** (low)
IgA	**0,28 g/L** (low)	**0,31 g/l** (low)	**0,17 g/l** (low)	**0,19 g/l** (low)	Not performed	0,66 g/l (normal)
IgM	**0,15 g/L** (low)	**0,06 g/l** (low)	**0,15 g/l** (low)	**0,25 g/l** (low)	**0,35 g/l** (low)	**0,40 g/l**, (low)
Specific antibody titers against S. pneumoniae	Not performed	**Low**	**Low**	**Absent**	Not performed	**Low**
Specific antibody titers against H. influenzae	Not performed	**Low**	Present	Not performed	Not performed	Not performed
Antibody response upon vaccination with polysacharide vaccine against S. pneumoniae	Not performed	**Decreased**	**Decreased**	Vaccinated, but no response measured	Not performed	**Decreased**
Antibody response upon vaccination with polysacharide-protein conjugate vaccine against H. Influenzae	Not performed	**Decreased**	Not performed	Not performed	Not performed	Not performed
Other laboratory findings	Lymphopenia, thrombocytopenia		Normal total numbers of B- and T-cells	Lymphopenia	Thrombocytopenia, CD4+ T-cell penia, disturbed lymphocyte response to mitogens	Thrombocytopenia, lymphopenia (low B-cells, CD4+ T-cells, switched memory and marginal zone B-cells)

This table summarizes clinical and immunological laboratory findings in 6 patients with Jacobsen syndrome described in literature [8–12]. Results depicted in bold represent abnormal findings when compared to healthy controls. In between brackets interpretation of measured values when compared to healthy controls is described

*IgG* immunoglobulin, *G, IgA* immunoglobulin A, *IgM* immunoglobulin M

In summary, these cases suggest that a (primary) immunodeficiency is a clinical feature in patients with JS.

In the current study, we evaluated a series of 6 patients that were randomly recruited from the Dutch JS patient network with a genetically confirmed diagnosis of JS for the evaluation of a potential immunodeficiency state. Medical records were studied to determine whether these patients suffered from recurrent infections. In addition, immunological analyses were performed. We were particularly interested to see whether an immunodeficiency state is a common feature of JS as suggested by previously published case reports. If an immunodeficiency state were found to be a common feature in JS, then its evaluation should be performed in all JS patients.

## Material and Methods

### Patients

This study was conducted in collaboration with the European Chromosome 11 Network (http://11q.chromosome11.eu). The Jacobsen syndrome patients were informed about the present study by members of this network. The 12 known Dutch members with a proven deletion of the long arm of chromosome 11 were addressed of which 6 patients were included in this study after obtaining written informed consent by them or their parents. For this study, Institutional Review Board approval was obtained (MEC-2013-026, Erasmus MC, Rotterdam, The Netherlands) and the study was performed according to the Declaration of Helsinki. Table 2 shows the demographic and genetic data from the included JS patients.

**Table 2:** Age, gender and genetic defects in our 6 patients with Jacobsen syndrome

Patient number	Age (years)	Gender	Genetic abnormalities	Clinical findings
1	24	M	11q- (45, x, ish der) (11) t (y;11) (p11.2;q24.1) (wcpyt)2	Recurrent otitis, sinusitis, upper and lower respiratory tract infections
2	35	F	Deletion 11q (q23.3 – qter)	No recurrent infections
3	14	F	Deletion 11q (q.24.1-qter)	Recurrent otitis, upper and lower respiratory tract infections
4	14	M	Deletion 11q 14.2 – 11q 22.2	Recurrent upper and lower respiratory tract infections
5	6	F	Deletion 11q (q23.3 – qter)	Recurrent otitis, sinusitis, upper and lower respiratory tract infections
6	10	F	Deletion 11q (q23.3 – qter)	Recurrent otitis, sinusitis, upper and lower respiratory tract infections

This table shows the age, gender (F = female, M = male) and confirmed genetic abnormalities of the 6 Dutch patients with Jacobsen syndrome studied

### Medical History

Patients were invited to visit the Clinical Immunology Outpatient Clinic at the Department of Internal Medicine, alone or with (one of) their parents. Medical history was evaluated in order to gain insight in the rate, site and severity of infections in the past. Also the needs for antibiotic treatment and hospital admission for severe infection were recorded.

### Blood Analysis

Blood samples were analysed for serum levels of IgG, IgG-subclasses, IgA and IgM. Total numbers of B lymphocytes, B lymphocyte subsets, T lymphocytes and natural killer (NK) cells were determined by flowcytometry as previously described [16]. Specific antibody titres against *S. pneumoniae* were analysed using a Luminex assay. The protocol used in our laboratory was adapted from the protocol as previously published by Borgers and co-workers [17].

### Vaccination Response

As part of the clinical workup for recurrent infections patients were immunized using a polysaccharide vaccine against *S. pneumoniae* (Pneumovax). All patients expect the one patient (patient 2) with pre-vaccination protective titers, i.e., 0.35 μg/ml in at least 7 out of 13 measured serotypes (type 1,3,4,5,6A,6B,7F,9V,14,18C,19A,19F,23F) received Pneumovax vaccine and 4 to 6 weeks after immunization specific antibody titers against S. pneumonia were measured and compared to pre-immunization titres. Using a Luminex assay, an at least 4-fold increase in titres reaching at least 1.00 μg/mL in at least 7 of the 13 measured serotypes 4 to 6 weeks after immunization was determined adequate [18, 19] using a Luminex assay.

## Results

### Clinical Evaluation

In the present study we evaluated 6 patients with a genetically confirmed diagnosis of JS (see Table 2) for an immunodeficiency. Five out of our 6 patients (all but patient number 2 in Table 2) suffered from recurrent upper and lower respiratory tract infections since early childhood (otitis, sinusitis and pneumonia) for which repetitive antibiotic treatments and/or hospital admissions were required. Although only few recorded data over the past years were available, amongst others *H. influenzae* and *S. pneumoniae* have been cultured in a number of infectious episodes in these patients. There were no patients that suffered from recurrent skin, gastrointestinal tract, or urinary tract infections. Patient 1 however, suffered from multiple and extensive warts on hands and feet since approximately 3 years, for which he had received local treatment, without discernible effect. There were no CT scans available from 5 out of 6 patients included, so we could not evaluate whether any of these patients suffered from bronchiectasis or other lung damage. The CT scan of one patient did not reveal any structural abnormalities of both lungs. Two patients were already on immunoglobulin supplementation therapy when analyzed. Patient 1 started intravenous immunoglobulin supplementation therapy once every 4 weeks in 2011 at the age of 23. Patient 6 started subcutaneous immunoglobulin replacement in 2010 at the age of 6. The frequency of infections decreased after immunoglobulin replacement therapy. There were no signs of auto-immune disease or lymphoproliferation in any of these patients. There was no history of malignant diseases.

### Initial Evaluation of the Immune System

The results on total numbers of B, T and NK cells and the levels of IgG, IgA and IgM are summarized in Table 3. In summary, 5 out of 6 patients showed low levels of IgG. Low IgA levels were found in 2 and low IgM levels in 5 of the patients. Total number of B cells were low in 4 out of 6 patients. Low numbers of T cells and NK cells were found in 2 and 4 patients, respectively. Age-adjusted normal values for IgG, IgA and IgM and total cell numbers are summarized in Supplemental Table 1.

**Table 3:** Immunological analysis of 6 patients with Jacobsen syndrome

	Patient 1	Patient 2	Patient 3	Patient 4	Patient 5	Patient 6
IgG (g/l)	**5,8**	*7,5*	**5,2**	**3,1**	**3,2**	**3,5**
IgA (g/l)	**0,45**	*1,89*	*0,65*	**0,30**	*0,88*	*0,61*
IgM (g/l)	**<0,30**	**<0,30**	*0,97*	**<0,30**	**0,17**	**<0,30**
Total number of B-cells (× 109 / l)	*0,10*	**0,05**	*0,22*	**0,16**	**0,09**	**0,07**
Total number of T-cells (× 109 / l)	*0,82*	**0,64**	*1,38*	*1,18*	*0,77*	**0,48**
Total number of NK-cells (× 109 / l)	**0,04**	**0,08**	*0,23*	*0,18*	**0,04**	**0,04**
Specific antibody titers against S. pneumoniae	**Low**	*Normal*	**Low**	**Low**	**Low**	**Low**
Antibody response upon vaccination with polysacharide vaccin against S. pneumoniae	**Decreased**	*Not performed*	**Decreased**	**Decreased**	**Decreased**	**Decresed**

This table shows total numbers of B, T and NK cells (all × 10^9^ cells / l) and levels of IgG, IgA and IgM (all in g/l) in 6 patients with Jacobsen syndrome. All values have been compared to normal values for age and are depicted in italic when normal for age and in bold when low for age. In supplementary table 1, age-related normal values for total cell numbers and immunoglobulin levels have been summarized

### Specific Antibody Titres

Five out of 6 patients (patient 1,3,4,5,6) showed inappropriate responses against Pneumovax. All of these 5 patients also had low total IgG levels (Table 3).

### B Lymphocyte Subset Analysis

**Fig. 1:**
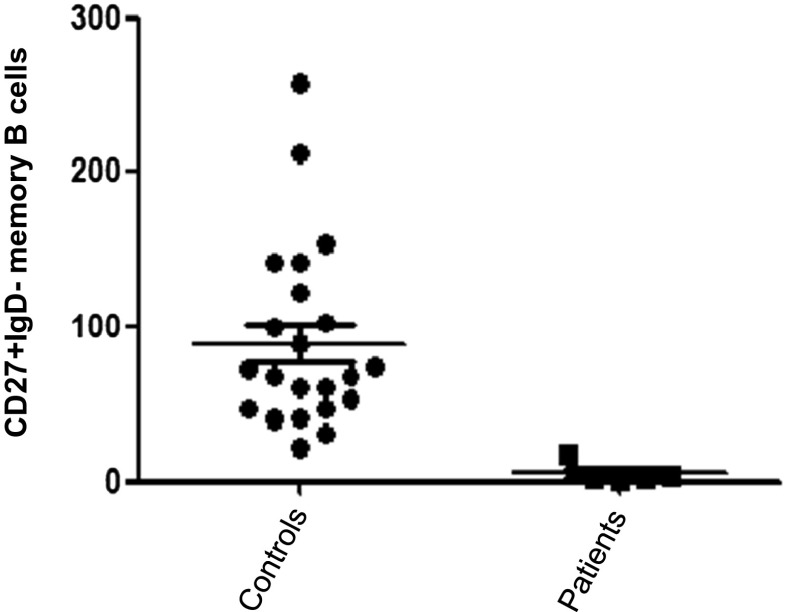
Total numbers of memory B cells in patients with Jacobsen syndrome compared to healthy controls. This figure shows the total numbers of memory B cells in patients with Jacobsen syndrome (*n* = 6) when compared to the number of memory B cells in healthy individuals (*n* = 20). A significant (*p* < 0.05) lower number of memory B cells is found in the patients with Jacobsen syndrome

By flowcytometry we investigated in more detail the B cell subsets (Table 4). We determined the absolute numbers of transitional B-cells (here defined as CD38^high^/CD24^high^); naive mature B-cells (CD38^dim^/CD24^dim^/IgD^+^/CD27^−^); marginal zone/natural effector B-cells (CD38^dim^/IgD^+^/CD27^+^) and memory B-cells (CD38^dim^/IgD^−^/CD27^+^) [20]. As shown in Table 3, the total number of B cells was low in 4 out of 6 patients studied. In more detail we found a decrease in the number of memory B cells in 5 out of 6 JS patients. In addition, marginal zone like B cells were decreased in 4 patients. Also the total numbers of CD4+ and CD8+ T cells were low in 1 and 2 patients, respectively. NK cells were below normal values in 4 patients. Importantly, CD38^dim^CD27+IgD- memory B cells in patients with JS were extremely low when compared to healthy controls (Fig. 1, *p* < 0.05).

**Table 4:** Analysis of B and T cell subsets in patients with Jacobsen syndrome

	Patient 1	Patient 2	Patient 3	Patient 4	Patient 5	Patient 6
	Total number of cells, (normal values adjusted for age)	Total number of cells, (normal values adjusted for age)	Total number of cells, (normal values adjusted for age)	Total number of cells, (normal values adjusted for age)	Total number of cells, (normal values adjusted for age)	Total number of cells, (normal values adjusted for age)
B-lymphocyte subsets (cells/μl)						
Transitional B-cells (CD38high/CD24high)	11, (3–50)	6, (3–50)	33, (4–108)	24, (4–108)	**10**, (11–77)	24, (11–77)
Naive mature B-cells (CD38dim/CD24dim/IgD+/CD27-)	69, (57–447)	33, (57–447)	96, (87–390)	114, (87–390)	**69**, (111–486)	**39**, (111–486)
Marginal zone/Natural effector B-cells (CD38dim/IgD+/CD27+)	**8**, (9–88)	**2**, (9–88)	62, (7–90)	12, (7–90)	**3**, (15–88)	**1**, (15–88)
Memory B-cells (CD38dim/IgD-/CD27+)	**2**, (13–122)	**2**, (13–122)	17, (10–76)	**4**, (10–76)	**5**, (13–100)	**1**, (13–100)
T-lymphocyte subsets (× 109/L)						
CD4+ T-lymphocytes	**0,2** (0.3–1.4)	0,5 (0.3–1.4)	0,9 (0.4–2.1)	0,7 (0.4–2.1)	0,4 (0.3–2.0)	0,3 (0.3–2.0)
CD8+ T-lymphocytes	0,6 (0.2–1.2)	**0,1** (0.2–1.2)	0,4 (0.2–1.2)	0,4 (0.2–1.2)	0,3 (0.3–1.8)	**0,2** (0.3–1.8)

Total numbers of various B cell and T cell subsets are presented for the 6 patients with JS. Numbers of cells are presented in cells/μl. Bold numbers indicate values that are lower than values in age-related healthy control values. In between brackets absolute numbers of cells for age-related healthy controls are shown

## Discussion

In the present study 6 patients with confirmed JS were evaluated for the presence of an immunodeficiency state. These patients had low IgG levels and impaired response to *S. pneumoniae* polysaccharide vaccination, defining a specific polysaccharide antibody deficiency. We also found a reduction in the number of memory B cells. These features are compatible with a CVID phenotype. The presence of a CVID phenotype in JS patients was previously demonstrated in sporadic cases as well (8–10, Table 1). Moreover, in our studies we demonstrated that T and NK cell numbers were low in a number of JS patients. Although no clinical features were found compatible with a T cell deficiency, it was recently reported that a JS patient with a T cell deficiency suffered from recurrent viral infections [11]. Recently, newborn screening for severe combined immunodeficiency (SCID) was introduced in several countries [21]. SCID is characterized by a low number of naïve T-cells and in newborn screening T-cell receptor excision circle (TREC) assay is used to detect T-cell lymphopenia [22]. Using this approach, patients with JS were identified shortly after birth, based on abnormal findings of low TRECS in these patients [21]. These and our findings of T cell deficiency in 2 out of 6 JS patients suggest that T cell deficiency may be also a common finding in JS. Finally, we demonstrated that 4 out of 6 patients had low numbers of NK cells. Only one patient (patient 1) suffered from infections (extensive human papilloma virus-induced warts) that may be due to a NK cell deficiency. In a previous report, genital and cutaneous condylomata were described in a patient that showed NK lymphopenia [12]. The presence of B, T and NK cell abnormalities and recurrent bacterial and viral infections is compatible with a combined immunodeficiency phenotype, present in JS.

Amongst the most reported infections are otitis and sinusitis. These were previously thought to be due to abnormal facial anatomy and potential chronic Eustachian tube dysfunction, which is also seen in other genetic syndromes, like trisomy of chromosome 21 (Down syndrome) [23]. However, over the past decades abnormalities in the blood B and T cell compartments, decreased IgM, IgG2 and IgG4 levels and poor immunoglobulin response to vaccinations have been demonstrated in Down syndrome [24]. Down syndrome is therefore recognized as a SPID and in recent years various other SPIDs have been identified, amongst others diGeorge syndrome [25, 26]. These SPIDs are disorders in which not only the immune system but also other organ systems are affected and in contrast to other primary immunodeficiencies, other features than the immune defects are the presenting symptoms [27]. We propose that JS should also be considered as a SPID, based on the susceptibility to infections as well as the immune cell defects described.

Therefore, close attention should be paid to immune cell function in these patients and when they present with recurrent infections immunological evaluation is warranted. It might be that more patients with JS require immunoglobulin replacement therapy than currently acknowledged. One of the important remaining questions is which genetic defect is responsible for the combined immunodeficiency state in JS. In previous studies an important role for ETS-1, a member of the ETS family of transcription factor, was proposed in cardiac abnormalities in Jacobsen patients. In mice deletion of ETS-1 leads to membranous ventricular septal defects, bifid cardiac apex and a non-apex-forming left ventricle [28]. Moreover, it was previously found that ETS-1, which is highly expressed in NK cells, B and T lymphocytes in physiological conditions, is involved in NK cell development, B cell differentiation and T lymphocyte development [29–33]. Several aberrations in B lymphocyte differentiation have been described in ETS-1 knockout mice, like enhanced differentiation into IgM- and IgG-secreting cells plasma cells and decreased titres of IgG2a [34, 35]. In mice expressing only one of the 2 known isoforms of ETS-1 diminished spleen cellularity including fewer memory cells was found [36]. ETS-1 knockout mice also have a variety of defects in T cell lineage, including aberrant thymic differentiation, reduced peripheral T cell numbers, reduced IL-2 production and impairments in Th1 and Th2 cytokine production [34]. Finally, in ETS-1 deficient mice lower numbers of NK cells and lower NK progenitors in the bone marrow are found [37, 38]. Interestingly, in our study, besides low numbers of memory B cells, low numbers of total NK cells and T lymphocytes were detected in a number of patients. This could be explained by a deletion of ETS-1. On the other hand, Friend Leukemia virus Integration-1 (FLI-1), which belongs to the ETS transcription factor family, is located on the long arm of chromosome 11 as well and is mainly expressed in haematopoietic cells. Loss of normal FLI-1 activation in mice resulted in significantly fewer splenic follicular B cells, an increased number of transitional and marginal zone B cells when compared to control mice [39]. There are no studies that have evaluated the role of FLI-1 on immune cell functions in human. In myeloid malignancies deletion of 11q was described. Itwas demonstrated that this deletion existed both as a sole abnormality and in association with other changes, including t(8;21) [40]. It could therefore be hypothesized that deletions in the long arm of chromosome 11 in JS coincides with defects on other chromosomes that are responsible for the immune cell defects described. In ongoing research we will further characterize which genes in the deletion are responsible for the immune defects in patients with JS. A larger (international) cohort of JS patients with known genetic defects will be analysed for immunodeficiencies. We will define the smallest common deletion in JS patients with proven immunodeficiency, to narrow the potential genes involved in the development of immune cell defects in JS. ETS-1 may be a candidate gene, but also FLI-1 could play a role and novel genes with currently unknown function might be identified as being potentially involved in immune cell function. The role of FLI-1 on immune cell development and function will be studied in more detail. Finally, the potential involvement of genetic defects affecting other chromosomes should be studied in more detail in JS.

In conclusion, we described for the first time a series of JS patients with a clinical picture and immunological abnormalities compatible with a combined immunodeficiency. Based on our findings and previously reported single cases we propose that JS should be considered as a SPID and as such should be added to the IUIS classification of primary immunodeficiencies [41]. Therefore, immunological screening should be performed in all patients with a confirmed diagnosis of JS. Early recognition of immunodeficiency leads to earlier therapeutic intervention, which results in prevention of recurrent infections and subsequent organ damage.

## Electronic supplementary material

Supplementary table 1 Normal values for immunoglobulin levels, B-, T- and NK-cells. This table shows normal values for immunoglobulins G, A and M as well as total numbers of B, T and NK cells. The left column shows normal values for adult patients >16 years of age, middle column aged 10–16 and right column aged 5–10. Immunoglobulin levels are presented in grams per liter and total cell numbers in cells per liter (PPTX 62 kb)
