# Supplementary material for: The 11q Terminal Deletion Disorder Jacobsen Syndrome is a Syndromic Primary Immunodeficiency
Source: J Clin Immunol. 2015 Nov 14;35(8):761–8. doi: 10.1007/s10875-015-0211-z (PMC4659842; doi:10.1007/s10875-015-0211-z)
Supplement: Supplementary file 1 — Normal values for immunoglobulin levels, B-, T- and NK-cells. This table shows normal values for immunoglobulins G, A and M as well as total numbers of B, T and NK cells. The left column shows normal values for adult patients >16 years of age, middle column aged 10–16 and right column aged 5–10. Immunoglobulin levels are presented in grams per liter and total cell numbers in cells per liter (PPTX 62 kb) [file 10875_2015_211_MOESM1_ESM.pptx]

## Slide 1
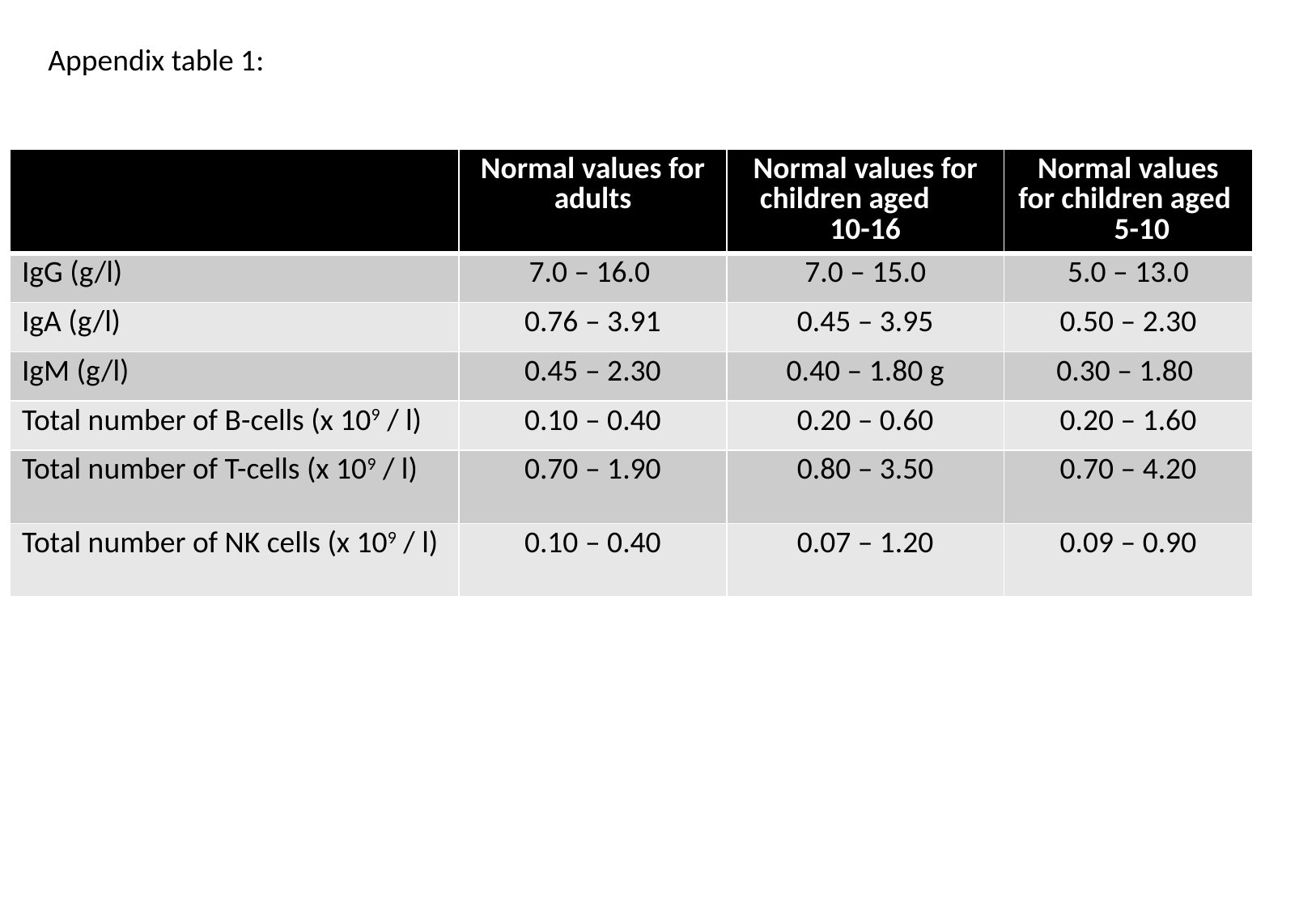

Appendix table 1:
| | Normal values for adults | Normal values for children aged 10-16 | Normal values for children aged 5-10 |
| --- | --- | --- | --- |
| IgG (g/l) | 7.0 – 16.0 | 7.0 – 15.0 | 5.0 – 13.0 |
| IgA (g/l) | 0.76 – 3.91 | 0.45 – 3.95 | 0.50 – 2.30 |
| IgM (g/l) | 0.45 – 2.30 | 0.40 – 1.80 g | 0.30 – 1.80 |
| Total number of B-cells (x 109 / l) | 0.10 – 0.40 | 0.20 – 0.60 | 0.20 – 1.60 |
| Total number of T-cells (x 109 / l) | 0.70 – 1.90 | 0.80 – 3.50 | 0.70 – 4.20 |
| Total number of NK cells (x 109 / l) | 0.10 – 0.40 | 0.07 – 1.20 | 0.09 – 0.90 |
